# Identification, Formation, and Occurrence of Perlolyrine: A β-Carboline Alkaloid with a Furan Moiety in Foods

**DOI:** 10.1021/acs.jafc.3c03612

**Published:** 2023-08-31

**Authors:** Tomás Herraiz, Adriana Peña, Antonio Salgado

**Affiliations:** † Spanish National Research Council (CSIC), Instituto de Ciencia y Tecnología de Alimentos y Nutrición (ICTAN-CSIC), José Antonio Novais 6, Ciudad Universitaria, 28040 Madrid, Spain; ‡ Centro de Espectroscopía de RMN (CERMN), Universidad de Alcalá (UAH), Campus Universitario Ctra. Madrid-Barcelona km 33.6, 28805 Alcalá de Henares, Madrid, Spain

**Keywords:** perlolyrine, β-carboline alkaloids, tryptophan, 3-deoxyglucosone, fructose, glucose, sucrose, Maillard reaction, foods

## Abstract

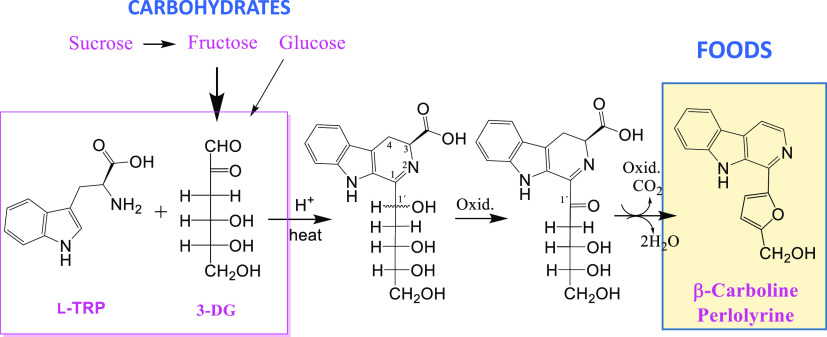

β-Carbolines are naturally occurring bioactive alkaloids found in foods and *in vivo*. This research reports the identification, characterization, mechanism of formation, and occurrence of perlolyrine (1-(5-(hydroxymethyl)furan-2-yl)-9*H*-pyrido[3,4-*b*]indole), a β-carboline with a furan moiety. Perlolyrine did not arise from l-tryptophan and hydroxymethylfurfural but from the reaction of l-tryptophan with 3-deoxyglucosone, an intermediate of carbohydrate degradation. The mechanism of formation occurs through 3,4-dihydro-β-carboline-3-carboxylic acid intermediates (imines), followed by the oxidation of C_1′_-OH to ketoimine and oxidative decarboxylation at C-3, along with dehydration and cyclization to afford the β-carboline with a furan moiety. The formation of perlolyrine was favored in acidic conditions and temperatures in the range of 70–110 °C. Perlolyrine occurred in the reactions of tryptophan with carbohydrates. The formation rate from fructose was much higher than from glucose. Sucrose also gave perlolyrine under acidic conditions and heating. Perlolyrine was identified in many foods by HPLC-MS and analyzed by HPLC-fluorescence. It occurred in many processed foods such as tomato products including tomato puree, fried tomato, ketchups, tomato juices, and jams but also in soy sauce, beer, balsamic vinegar, fruit juices, dried fruits, fried onion, and honey. The concentrations ranged from an undetected amount to 3.5 μg/g with the highest average levels found in tomato concentrate (1.9 μg/g) and soy sauce (1.5 μg/mL). The results show that perlolyrine formed during the heating process of foods. It is concluded that perlolyrine is widely present in foods and it is daily ingested in the diet.

## 1 Introduction

**Figure 1:**
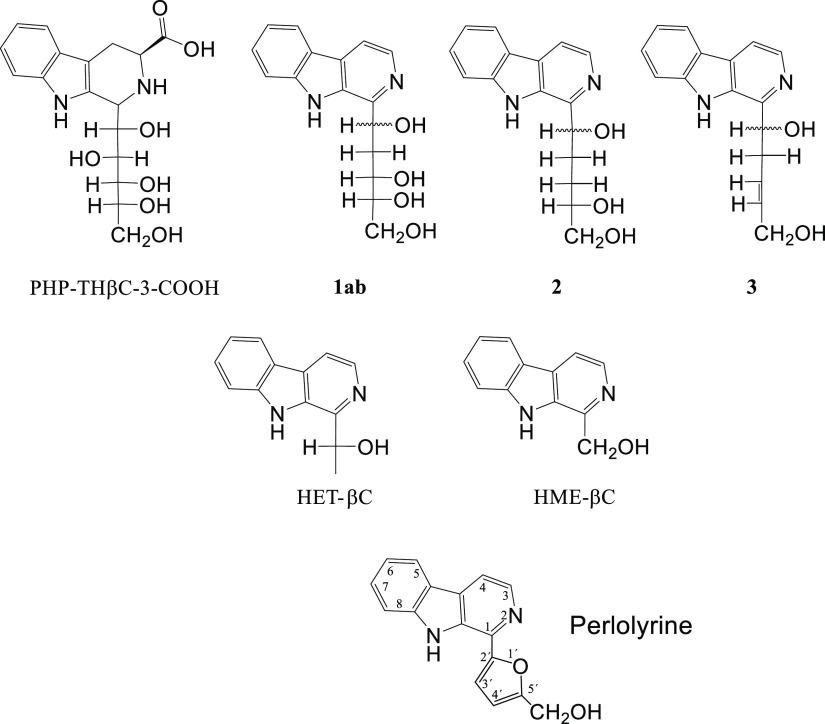
Structures of tetrahydro-β-carbolines and aromatic βCs derived from carbohydrates^16,23,28^ and the βC perlolyrine containing a furan moiety.

β-Carboline (9*H*-pyrido[3,4-*b*]indole) alkaloids (βCs) are bioactive indole compounds occurring in foods, plants, and biological fluids and tissues.^1−4^ They exert effects on the central nervous system (CNS) by the interaction with the serotonin uptake system, benzodiazepine receptors, and imidazoline binding sites and inhibit enzymes such as monoamine oxidase (MAO) and kinases.^3,5,6^ βCs exhibit antidepressant and behavioral effects associated with changes in neurotransmitter levels and inhibition of MAO^7−11^ and can exert neuroprotective/neurogenesis actions.^12^ These alkaloids can be also bioactivated by *N*-methylation, affording endogenous neurotoxins (i.e., β-carbolinium cations) that resemble the MPTP neurotoxin.^3^ βCs can be comutagenic compounds, bind to DNA, and react with hydroxyl radical (OH^•^).^13,14^ Therefore, β-carbolines exert many biological, pharmacological, and toxicological activities, and their presence in foods and *in vivo* is a matter of interest. β-Carboline alkaloids can be classified as tetrahydro-β-carbolines (THβCs) or aromatic β-carbolines (βCs). THβCs are produced through the Pictet–Spengler reaction from tryptophan or indoleethylamines and carbonyl compounds (aldehydes or α-keto acids).^1^ THβCs coming from tryptophan and aldehydes afford tetrahydro-β-carboline-3-carboxylic acids (THβC-3-COOH) that abound in foods with concentrations reaching up to 500 mg/L.^2,15^ THβCs can be also formed from carbohydrates. Thus, the Pictet–Spengler reaction among tryptophan and glucose affords the THβC pentahydroxypentyl-tetrahydro-β-carboline-3-carboxylic acid (PHP-THβC-3-COOH)^16,17^ (Figure 1). PHP-THβC-3-COOHs have been reported in foods, and relatively high amounts found in tomato products, fruit juices, and jams.^16^ This compound was also found in human urine.^18,19^ Aromatic βCs generally arise from their corresponding THβCs through oxidation.^20,21^ Thus, the βCs norharman and harman, which are generated in many foods, including meats and fish during cooking, are produced from the oxidation of THβC-3-COOH.^20,22^ Aromatic βCs derived from carbohydrates (**1ab–3**) have been found in foods and natural products^1,23−27^ (Figure 1). These compounds arise from the reactions of tryptophan and fructose or sucrose after hydrolysis, and also glucose in a minor extent, but they do not arise from the oxidation of the corresponding THβCs.^23^ Recently, the mechanism of formation of carbohydrate-derived βCs (**1ab–3**) has been demonstrated as coming from the reaction of tryptophan with 3-deoxyglucosone.^23,28^ Moreover, new molecules of βCs arising from 3-deoxyglucosone and the α-dicarbonyl compounds, glyoxal and methylglyoxal, which arise from the degradation of carbohydrates during glycation processes, have been characterized, identified, and quantified in foods^28^ (Figure 1). These βCs could be considered a class of advanced glycation end products (AGEs). AGEs form from the degradation of carbohydrates during glycation processes *in vivo* and could play a role in human diseases such as diabetes and neurodegenerative and cardiovascular diseases,^29,30^ whereas it is unknown whether those actions are extensible to food-derived AGEs.

Perlolyrine is an aromatic βC containing a furan moiety that has been previously isolated from natural products^31−33^ and soy sauce^33,34^ (Figure 1), but its mechanism of formation as well as the origin of the furan moiety remains unknown. Perlolyrine shows interesting biological activities. It is a chemopreventive agent that induces phase II enzymes^34^ and an antiproliferative agent against tumor cells.^35^ However, very little is known about its possible formation, presence, and significance in foods. This research was aimed to study the formation and presence of perlolyrine in foods. It describes for the first time the factors influencing the formation of this βC and highlights the mechanism of formation as arising from 3-deoxyglucosone, an intermediate of the degradation of carbohydrates, and not from hydroxymethylfurfural, as it was thought. Perlolyrine was formed in the reactions of tryptophan with fructose and glucose. Moreover, perlolyrine was identified and quantified in many foods, most of them, for the first time. It is concluded that perlolyrine is widespread in foods, where it is formed during food processing and cooking, and therefore, this βC is daily ingested during food consumption.

## 2 Materials and Methods

### 2.1 Chemical Compounds and Foods

Commercial samples of foods were purchased in local supermarkets and used as supplied. l-Tryptophan, d-(−)-fructose, and 5-(hydroxymethyl)furfural (5-HMF) were obtained from Sigma (St. Louis, MO, USA), d-(+)-glucose monohydrate was obtained from Merck (Darmstadt, Germany), sucrose was obtained from Scharlau (Barcelona, Spain), and 3-deoxy-d-glucosone (3-deoxyglucosone (3-DG)) from Biosynth-Carbosynth (Compton, UK). The βC perlolyrine was isolated from reactions of l-tryptophan and d-fructose, as indicated below, and characterized by NMR and mass spectrometry.

### 2.2 Preparation, Isolation, and Characterization of Perlolyrine

l-Tryptophan (600 mg) and d-fructose (2000 mg) dissolved in 40 mL of 100 mM phosphate buffer (pH 3) were reacted at 80 °C for 96 h and extracted in alkaline pH (pH 9.5–10) with dichloromethane. The solvent was evaporated in a rotary evaporator, dissolved in acidic water (pH 2), and loaded into a classic chromatographic column loaded with the C_18_ stationary phase that was eluted with deionized water with increasing percentages of acetonitrile (5%), and containing 0.5% formic acid. The fractions were analyzed by HPLC and HPLC-MS and those containing perlolyrine ([M + H]^+^ at *m*/*z* 265) were pooled and extracted again in alkaline pH with dichloromethane to afford 1-(5-(hydroxymethyl)furan-2-yl)-9*H*-pyrido[3,4-*b*]indole, 1-(5-hydroxymethyl-2-furyl)-β-carboline, or perlolyrine (also known as perlolidin, perlolyrin, or substance YS) (7.6 mg) (Figure 1) with a purity higher than 90% by HPLC. Spectral characterization was accomplished by ^1^H NMR, ^13^C NMR, COSY, HSQC, and HMBC experiments. NMR experiments were done in a Varian INNOVA 500 NMR system (Varian Inc., Palo Alto, CA, USA), equipped with a CHX ^1^H/^13^C/^15^N-^31^P probe head, a *z*-gradient module, and a variable temperature unit. ^1^H NMR (500 MHz, DMSO): δ (ppm) 11.19 (s, 1H), 8.37 (d, *J* = 5.1 Hz, 1H), 8.26 (d, *J* = 8.2 Hz, 1H), 8.07 (d, *J* = 5.1 Hz, 1H), 7.76 (d, *J* = 8.2 Hz, 1H), 7.60 (ddd, *J* = 8.4, 7.0, 1.2 Hz, 1H), 7.29 (ddd, *J* = 8.0, 7.1, 1.0 Hz, 1H), 7.21 (d, *J* = 3.3 Hz, 1H), 6.58 (d, *J* = 3.3 Hz, 1H), 5.44 (t, *J* = 6.2 Hz, 1H), 4.67 (d, *J* = 6.2 Hz, 2H). ^13^C NMR (126 MHz, DMSO): δ (ppm) 156.75 (furan C-2), 152.08 (furan-C5), 140.92 (C-9a), 138.17 (C-3), 133.13 (C-1), 130.46 (C-8a), 129.42 (C-4a), 128.41 (C-7), 121.63 (C-5), 120.62 (C-4b), 119.69 (C-6), 113.63 (C-4), 112.41 (C-8), 109.62 (furan C-4), 109.03 (furan C-3), 55.94 (CH_2_OH) (Table S1 and Figure S1). High-resolution ESI^+^ mass spectrometry (HRMS) (LC-MS-Q-TOF, Agilent G6530A) gave *m*/*z* 265.0966 [M + H]^+^ corresponding to the C_16_H_12_N_2_O_2_ molecular formula and a theoretical exact mass of 264.0899 g/mol (MH^+^ at 265.0977); mass error of 4.1 ppm. Mass spectra by MS/MS (20 V): *m*/*z* at 265.09, 206.08, 247.08, 219.08, 235.08. Those spectroscopic results are in good agreement with the reported spectral data of this substance.^36,37^

### 2.3 Formation of Perlolyrine in the Reactions of Tryptophan with 5-HMF, 3-DG, and Carbohydrates and Formation in Processed Foods

Model reactions of l-tryptophan and 5-HMF, 3-DG, or the carbohydrates glucose, fructose, and sucrose were carried out to evaluate the formation of perlolyrine. For that, solutions of l-tryptophan (0.5 mg/mL) and 5-HMF (0.1 mg/mL) were carried out in 100 mM phosphate buffer adjusted at different pHs (1.3, 3.1, 5, 7.4, and 9) and reacted for 2–4 h at 90 °C and subsequently analyzed by HPLC. Alternatively, solutions of l-tryptophan (0.5 mg/mL) and 5-HMF (0.1 mg/mL) in pH 3.1 were reacted at different temperatures (25 up to 130 °C) and analyzed by HPLC. On the other hand, l-tryptophan (0.5 mg/mL) and 3-DG (0.1 mg/mL) in 100 mM phosphate buffer adjusted at different pHs (1.3, 3.1, 5, 7.4, and 9) were reacted in a water bath at 90 °C for 2–4 h and analyzed by HPLC. Moreover, solutions of l-tryptophan (0.5 mg/mL) and 3-DG (0.1 mg/mL) in 100 mM phosphate buffer adjusted at pH 3.1 were reacted in glass tubes at different temperatures (25–130 °C) for 2–4 h and analyzed by HPLC. The reactions of tryptophan with carbohydrates were as follows: tryptophan solutions (0.5 mg/mL) with glucose (5 mg/mL), fructose (4.5 mg/mL), or sucrose (8.5 mg/mL) were reacted in 100 mM phosphate buffer adjusted at different pHs (1.3, 3.1, 5, 7.4, and 9) for 20 h at 90 °C. Reactions were also carried out in higher concentrations: tryptophan (2 mg/mL) and glucose (40 mg/mL), fructose (36.4 mg/mL), or sucrose (69.1 mg/mL) in 100 mM phosphate buffer adjusted at pHs 3.1, 5, and 7.4 were reacted at 80 °C for 20 h. In addition, solutions of l-tryptophan (2 mg/mL) and fructose (36.4 mg/mL) in 100 mM phosphate buffer (pH 2.9) were reacted at different temperatures (37–130 °C) for 20 h. All reactions were carried out at least in duplicate. Aliquots of the reactions were injected into the RP-HPLC and analyzed for perlolyrine by DAD and fluorescence detection and also by HPLC-MS. To study the mechanism of formation of perlolyrine, reactions of l-tryptophan with 3-DG or fructose previously preheated at 100 °C (2 h) were carried out at 70 °C for 4 h and analyzed by HPLC and HPLC-MS. The HPLC chromatographic peaks with an absorption maxima at around 355–375 nm (3,4-dihydro-β-carboline-3-carboxylic acids) corresponding to the HPLC fraction between 4.5 and 6 min were collected and pooled from successive HPLC injections and concentrated to dryness under vacuum (45 °C). Subsequently, it was dissolved in water, and aliquots of 200 μL were treated with SeO_2_ (4 mg), adjusted to pH 3, incubated at 70 °C for 3 h, and analyzed for the presence of perlolyrine while comparing with the corresponding controls.

To study the formation of perlolyrine in foods, several food samples were processed in the laboratory and compared with controls. Thus, commercial fresh tomato juice (not from concentrate) was heated at 90 °C in a water bath, or 110 °C for 5 h in an oven, and subsequently analyzed for perlolyrine, as indicated below; fresh tomatoes were crushed using an Ultra-Turrax homogenizer and heated at 110 °C for 5 h and analyzed for perlolyrine; and finally, commercial canned natural crushed tomato puree was heated at 90 °C for 5 h and analyzed for perlolyrine.

### 2.4 Isolation of Perlolyrine in Foods by Solid-Phase Extraction (SPE)

The βC perlolyrine was isolated from foods by SPE using propylsulfonic acid-derivatized silica PRS cartridges (Bond Elut, 500 mg, 3 mL volume, Agilent). Samples of solid foods (2–5 g) or liquid samples (5 mL) were added with 0.6 M HClO_4_ (15–20 mL), homogenized using an Ultra-Turrax homogenizer, and centrifuged at 10,000 rpm for 15 min at 0–5 °C. The conditioning of PRS columns was made with 6 mL of methanol and 6 mL of 0.1 M HCl. Aliquots (5 mL) were spiked with 0.5 mL of 1-ethyl-β-carboline solution (EβC) (0.08 mg/L) as an internal standard (IS) and subsequently loaded onto PRS columns using a vacuum manifold. After washing with deionized water (2 mL) and 3 mL of 0.4 M K_2_HPO_4_ (pH 9.1), perlolyrine was eluted with 3 mL of 0.4 M K_2_HPO_4_ (pH 9.1):methanol (1:1) and it was analyzed by HPLC-fluorescence and the presence of the compound confirmed by HPLC-MS. The performance of the SPE procedure gave recoveries of perlolyrine (40 μg/L) higher than 95% (*n* = 3), repeatability of 3% RSD (*n* = 3), and accuracy of 2.2% mean error (*n* = 3) after analysis by HPLC-FLD, as mentioned below. The LOD and LOQ values were 0.5 and 1.5 μg/L, respectively.

### 2.5 Chromatographic Analysis of Perlolyrine and Identification by HPLC-MS

Chromatographic analysis of perlolyrine in model reactions was carried out with a 1050 high-performance liquid chromatograph (Agilent Technologies) with a 1100 series DAD and a 1046A fluorescence detector. The analysis of perlolyrine isolated from foods was carried out using a 1200 series liquid chromatograph equipped with a 1200 series DAD and 1260 series fluorescence detectors (Agilent). A 150 mm × 3.9 mm, 5 μm, Novapak C18 column (Waters) was used for HPLC separation. The eluents were 50 mM ammonium phosphate buffer adjusted to pH 3 with 85% phosphoric acid (eluent A) and 20% eluent A in acetonitrile (eluent B). The gradient was set to 0% B to 32% B in 8 min and then 90% B in 12 min. The flow rate was 1 mL/min, the oven temperature was 40 °C, and the injection volume was 20 μL. Detection of perlolyrine was done by absorbance (DAD) and fluorescence in two conditions: 300 nm, excitation, and 433 nm, emission, and at 420 nm, excitation, and 460 nm, emission. The analysis of perlolyrine in the reactions was done from calibration curves built with perlolyrine using detection at 280 nm. Perlolyrine isolated from foods by SPE was analyzed by fluorescence detection that was programmed at 300 nm (excitation) and 433 nm (emission) for detection of the IS and modified at 9 min to 420 nm (excitation) and 460 nm (emission) for detection of perlolyrine. Quantitative analyses were obtained from calibration curves of the standard perlolyrine against the compound EβC used as an IS that were carried out through the entire SPE isolation procedure, as mentioned above. Identification of perlolyrine in the samples was accomplished by HPLC with DAD and fluorescence spectra recording of the chromatographic peaks and coelution with authentic standards and confirmed by HPLC-MS.

For identification purposes, SPE extracts were concentrated using a speed vacuum concentrator and analyzed by HPLC-MS (electrospray ionization mode, ESI^+^), as previously reported.^28^ The instrument used was a HPLC-MS Waters separations module Alliance e2695 with a quadrupole QDa Acquity and a Waters Photodiode Array Detector (PDA) 2996, working under positive electrospray ionization (ESI^+^), and equipped with a 2.1 × 100 mm, 3 μm, 100 Å, C18 Atlantis T3 column (Waters). Chromatographic separation was accomplished with a program containing the eluents A (water), B (ACN), and C (2% formic acid) under a gradient going from 5% B, 5% C, and 90% A to 90% B, 5% C, and 5% A in 18 min. A flow of 0.35 mL/min and an injection volume of 9 μL were used. The mass spectra were acquired in the ESI positive ion ionization mode at various cone voltages (10, 20, and 40 V) and at a mass range of 85–1250 amu.

## 3 Results

### 3.1 Isolation, Characterization, and Formation of Perlolyrine

**Figure 2:**
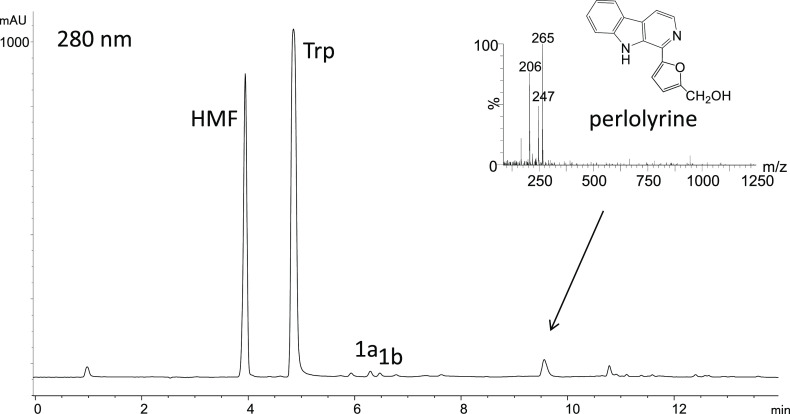
HPLC chromatogram (280 nm) of the reaction of 3-DG (0.1 mg/mL) with l-tryptophan (0.5 mg/mL) (90 °C, 4 h) and formation of perlolyrine. The mass spectrum of perlolyrine was obtained by HPLC-MS analysis of the reaction mixture.

**Figure 3:**
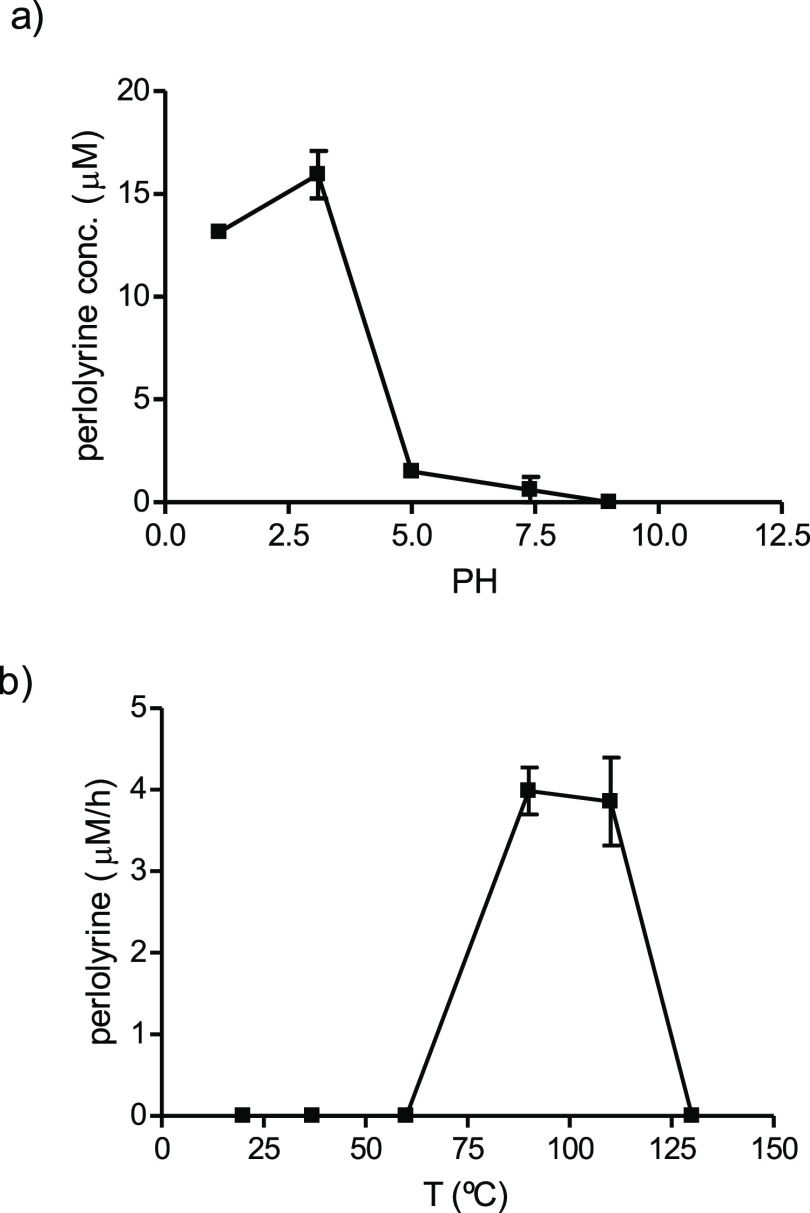
Formation of perlolyrine (μM) in the reactions of l-tryptophan (0.5 mg/mL) with 3-DG (0.1 mg/mL) as a function of pH (90 °C, 4 h) (a) and formation rate of perlolyrine (μM/h) in the same reactions as a function of temperature (pH 3.1) (b).

Crude reactions of l-tryptophan and d-fructose (pH 3.1) at 80 °C (Figure S2) were extracted with dichloromethane in alkaline pH. Evaporation of the organic phase and purification by column chromatography (C_18_) afforded a βC derivative that was characterized by NMR and MS as the βC containing a hydroxymethylfuran moiety at C-1, namely 1-(5-hydroxymethyl-2′-furyl)-β-carboline, also called perlolyrine (Figure 1). It has been previously assumed that perlolyrine arises from the reaction of 5-HMF with tryptophan.^38^ Then, we studied the formation of perlolyrine through a Pictet–Spengler reaction between tryptophan and 5-HMF in aqueous media. l-Tryptophan did not react with 5-HMF to give perlolyrine either at pHs ranging from 1.3 to 9 or temperatures from 25 to 110 °C. These results ruled out the formation of perlolyrine by the Pictet–Spengler reaction of tryptophan and 5-HMF, followed by oxidative decarboxylation. In contrast, l-tryptophan did react with the α-dicarbonyl compound 3-DG and gave the βC perlolyrine (Figure 2). The formation of perlolyrine from 3-DG occurred simultaneously to the formation of the carbohydrate-derived βCs, which also originated from this precursor:^23,28^ 1-(1,3,4,5-tetrahydroxypent-1-yl)-β-carboline isomers (**1a/b)**, 1-(1,4,5-trihydroxypent-1-yl)-β-carboline (**2**), and 1-(1,5-dihydroxypent-3-en-1-yl)-β-carboline (**3**) (Figure 1). The formation of perlolyrine from 3-DG and tryptophan was studied under different conditions of pH and temperature. Perlolyrine formation increased under acidic pH (optimum pH at 1–3) and with increasing temperatures up to 110 °C (Figure 3). However, the formation of this βC was not favored at higher temperatures like 130 °C. Additionally, physiological conditions of temperature (37 °C) and pH (7.4) did not lead to the formation of perlolyrine.

**Figure 4:**
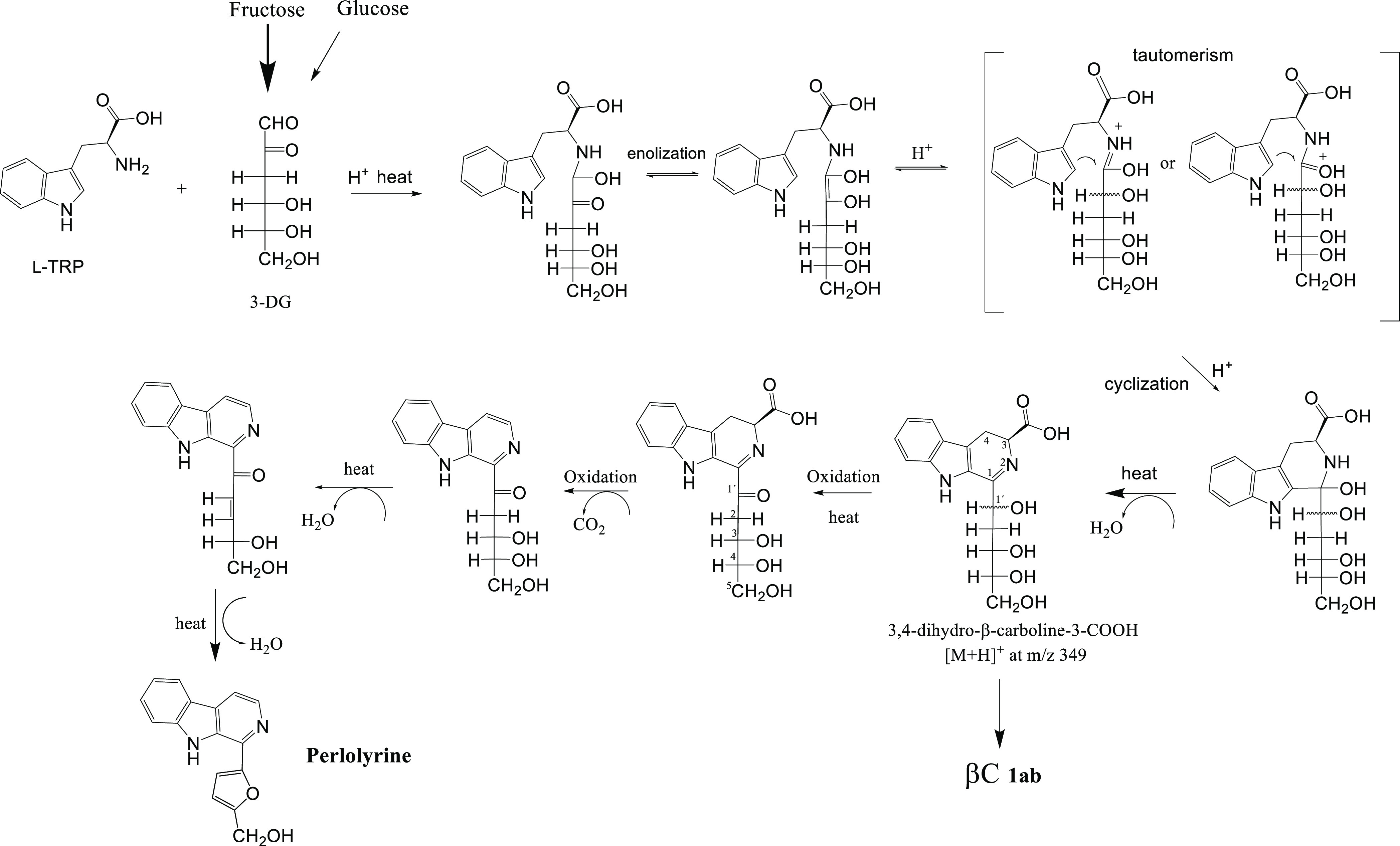
Mechanism proposed for the formation of perlolyrine from the reaction of l-tryptophan (l-TRP) with 3-DG arising from fructose and glucose degradation.

The formation of perlolyrine from tryptophan and 3-DG could follow the mechanism proposed in Figure 4. Initially, it follows the steps described before for carbohydrate-derived βCs and α-dicarbonyl-derived βCs.^23,28^ Tryptophan reacts with 3-DG, affording through enolization, tautomerism, and cyclization the 3,4-dihydro-β-carboline-3-carboxylic acid intermediates with C_1′_-OH in the carbohydrate moiety. These intermediates were detected in the reactions of tryptophan with 3-DG or preheated fructose, at 70 °C and short times (4 h) by HPLC-MS ([M + H]^+^ at *m*/*z* 349 and mass fragments at *m*/*z* 331 and 285), and absorbance spectra with a λ_max_ at 355–375 nm. They would be precursors of both perlolyrine and carbohydrate-derived βCs (**1ab**).^23,28^ To afford perlolyrine, the 3,4-dihydro-β-carboline-3-carboxylic acids (imines) with C_1′_-OH could be oxidized to C_1′_ = O and subsequently follow a process of oxidative decarboxylation to give the ring of βC and after dehydration, cyclization, and dehydration afford the furan ring of perlolyrine (Figure 4). This mechanism was supported by the results obtained here because the intermediates 3,4-dihydro-β-carboline-3-carboxylic acids with absorption maxima at 355–375 nm (fraction of 4.7–6 min in the HPLC) were isolated and afforded perlolyrine after oxidation with SeO_2_ and heating (Figure S3).

### 3.2 Identification, Formation, and Occurrence of Perlolyrine in the Reactions of l-Tryptophan with Carbohydrates

**Figure 5:**
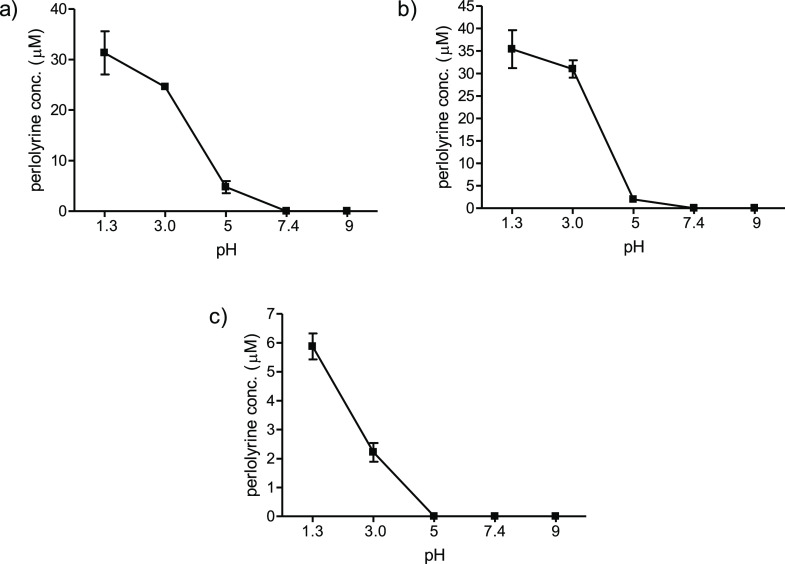
Formation of perlolyrine (μM) in the reactions of l-tryptophan 0.5 (mg/mL) with fructose (4.5 mg/mL) (a), sucrose (8.5 mg/mL) (b), or glucose (5 mg/mL) (c) as a function of pH (reactions at 90 °C, 20 h).

**Figure 6:**
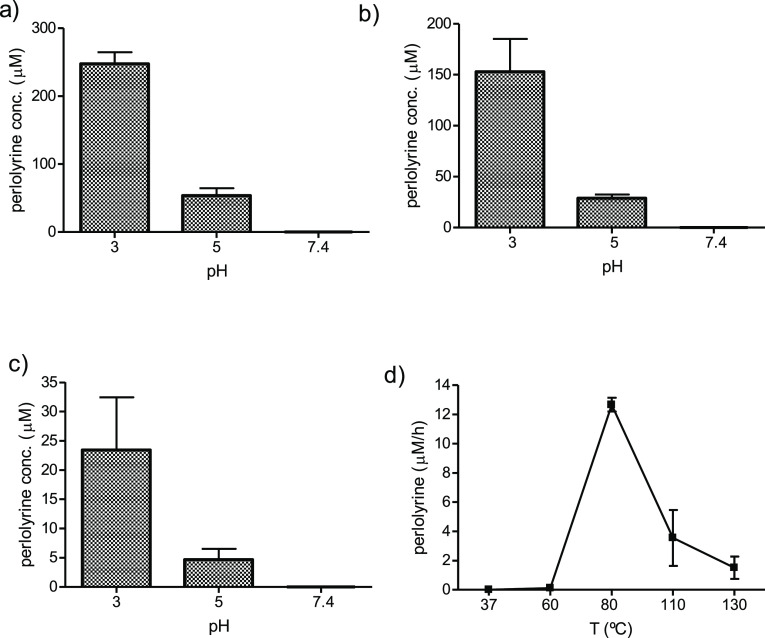
Formation of perlolyrine (μM) in the reactions of l-tryptophan (2 mg/mL) with fructose (36.4 mg/mL) (a), sucrose (69.1 mg/mL) (b), or glucose (40 mg/mL) (c) in different pHs (pHs 3–7.4) at 80 °C for 20 h and formation rate of perlolyrine (μM/h) in the reactions of l-tryptophan (2 mg/mL) with fructose (36.4 mg/mL) at different temperatures and pH 2.9 (20 h) (d).

Perlolyrine occurred in the reactions of l-tryptophan with fructose, sucrose, or glucose, and its presence was confirmed by HPLC-MS (Figure S4). The formation of perlolyrine from carbohydrates increased under acidic pHs, and it was higher for fructose and sucrose than for glucose (Figure 5). Perlolyrine increased in higher concentrations of tryptophan and carbohydrates (Figure 6), and its formation rate increased with the temperature, although higher temperatures such as 110 and 130 °C resulted in less amount of perlolyrine than lower temperatures such as 80 °C. Perlolyrine also increased in acidic pHs (pH 3), whereas pHs >5 did not favor the formation of this compound (Figure 6). The levels of perlolyrine were much higher in the reactions of tryptophan with fructose than with glucose. Under the same conditions, perlolyrine obtained from fructose was more than 10 times than that resulting from glucose (Figures 5 and 6). Under acidic conditions and heating (e.g., pH 1–3), the concentration of perlolyrine generated from sucrose was similar to that of fructose; however, it decreased at pH 5. The formation of perlolyrine from sucrose indicates that it was hydrolyzed, affording fructose, which was subsequently involved in the formation of perlolyrine, whereas glucose surely contributed to a minor extent.

### 3.3 Identification and Occurrence of Perlolyrine in Foods

**Figure 7:**
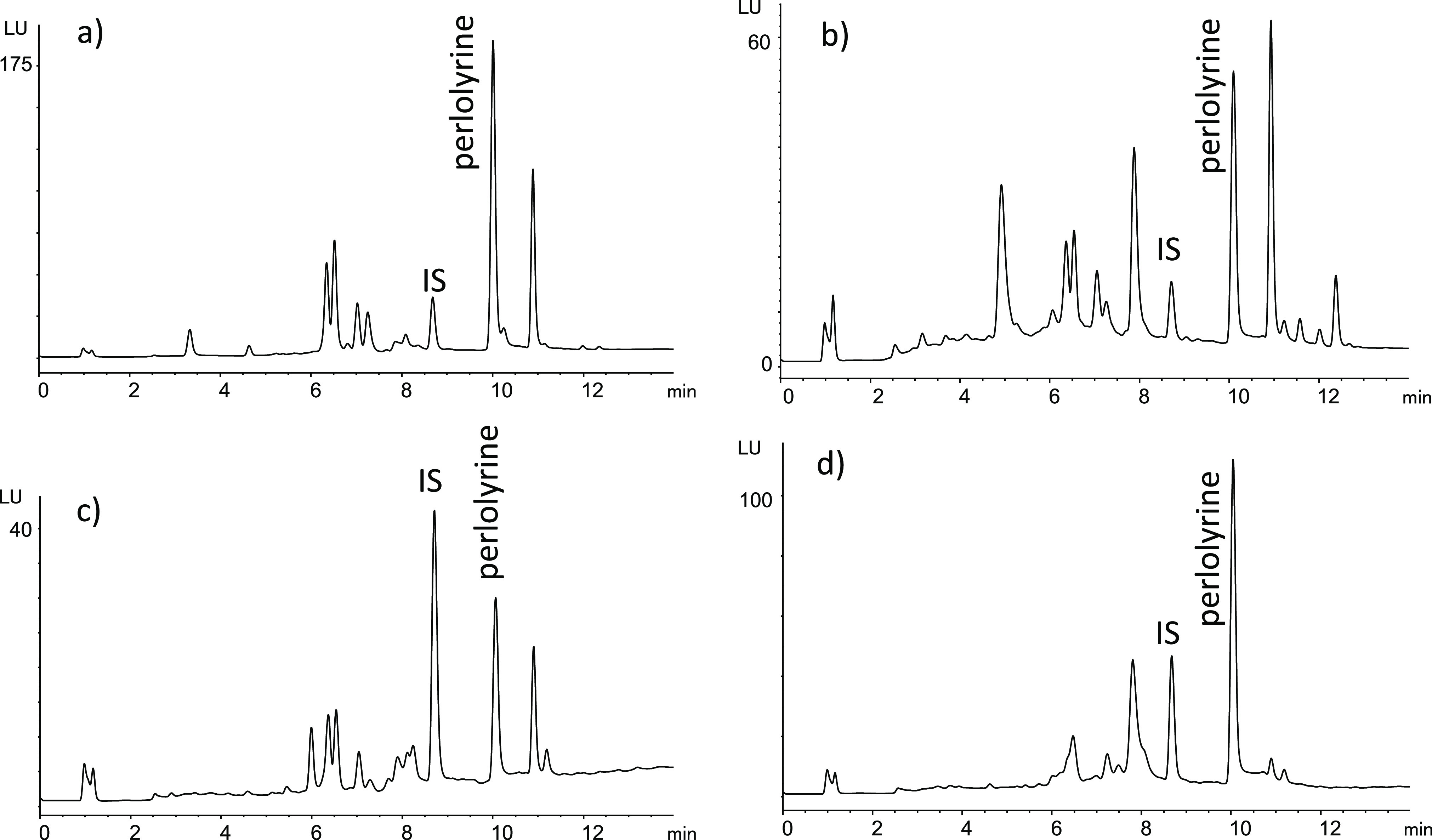
HPLC-FLD chromatograms of perlolyrine in representative food samples analyzed following isolation by SPE. (a) Tomato juice from concentrate, (b) soy sauce, (c) prunes, and (d) beer. Fluorescence detection: 300 nm, excitation, and 433 nm, emission (0–9 min); 420 nm, excitation, and 460 nm, emission (9–14 min). The relative responses in chromatograms vary with fluorescence gain used.

**Figure 8:**
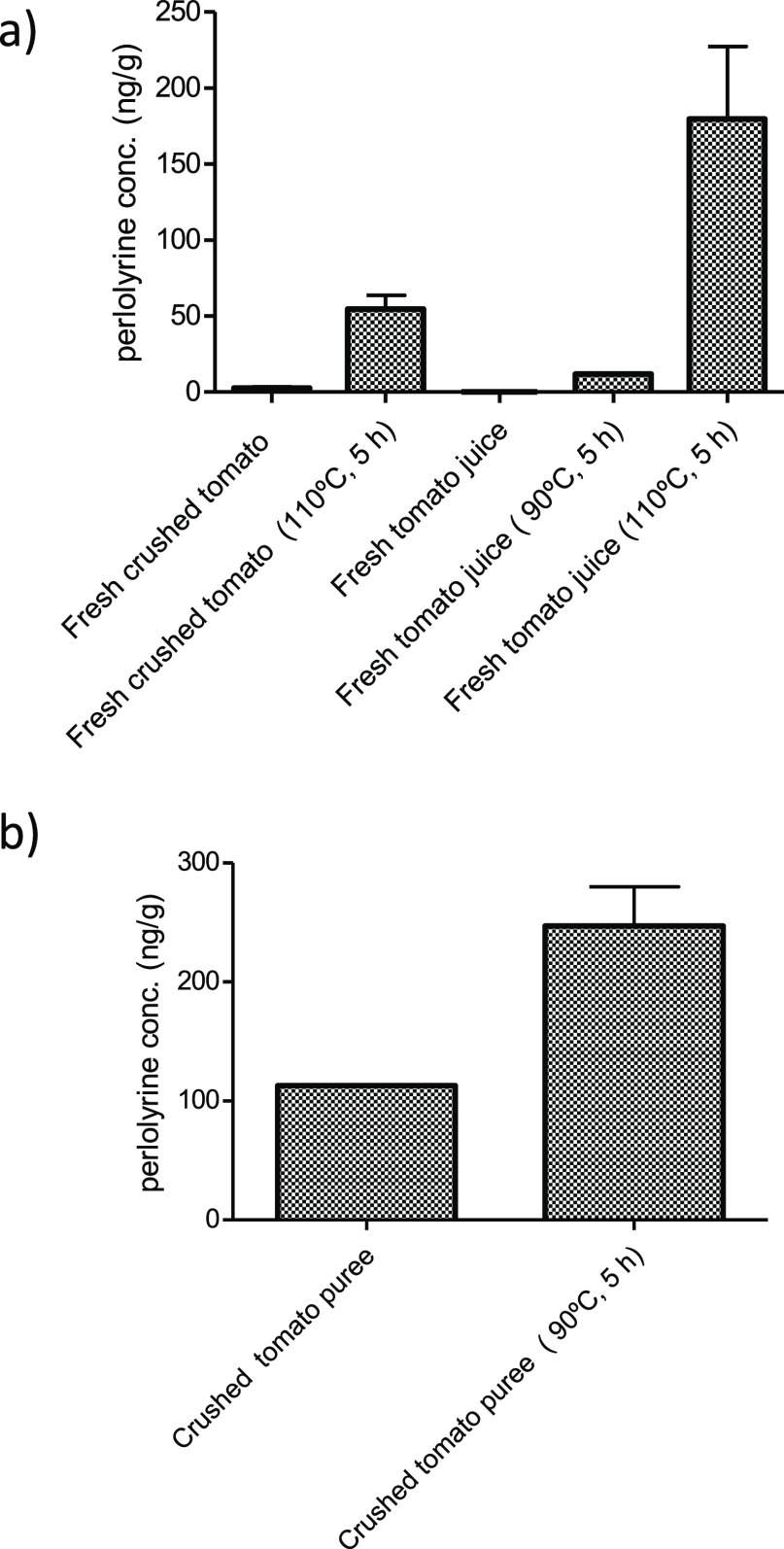
Formation of perlolyrine (ng/g) in foods processed by heating. (a) Fresh crushed tomato and commercial fresh tomato juice (not from concentrate) and the same samples after heating in the laboratory, and (b) formation of perlolyrine (ng/g) in commercial canned crushed tomato puree and the same sample after heating in the laboratory.

The presence of perlolyrine in foods was subsequently investigated by HPLC-MS following isolation by SPE (Figure S5). Perlolyrine was identified in many commercial foods including processed tomato products such as fried tomato puree, tomato juice, ketchup, tomato concentrate, and tomato jam but also in soy sauce, sauces, molasses, beer, fruit juices, dried fruits, and honey. Subsequently, perlolyrine was analyzed by HPLC with fluorescence detection (Figure 7), and its concentrations were determined in foods (Table 1). The occurrence of perlolyrine was widespread in the foods studied. Higher contents were found in processed tomato products such as fried tomato, ketchup, tomato juices, canned crushed tomato, and tomato jam with the highest level found in tomato concentrate (2455 ng/g). A highest level of 3483 ng/mL was reached in soy sauces. Moderate levels were encountered in barbecue sauce, balsamic vinegar, beer, and fruit juices made from concentrate juice such as grape and pineapple juice. Perlolyrine was also found in dried fruits such as prunes, raisins, and apricots and in fried onion and honey. Other processed foods analyzed such as cookies, cereals, and breads did not seem to contain perlolyrine or contained very low levels. The results in Table 1 indicate that perlolyrine occurred in processed foods. Indeed, the formation of this βC during processing was evidenced when fresh tomato juice and fresh crushed tomato samples were subjected to heating in the laboratory (Figure 8). Negligible amounts appeared in the fresh samples, but perlolyrine increased after heating. Moreover, the concentration of perlolyrine in commercial canned crushed tomato also increased after heating. This sample already contained perlolyrine likely owing to heating during the elaboration process.

**Table 1:** Concentration of the βC Perlolyrine in Commercial Foods

foods	*X*^f^ (ng/g^a^ or ng/mL^b^)	SD	range (ng/g or ng/mL)
fried tomato (5)^c^	551.2^a^	433.7	20.34–1101
ketchup (3)	394.7^a^	149.1	269.4–559.6
tomato juice from concentrate (4)	503.0^b^	331.5	145.2–796.3
tomato concentrate (paste) (3)	1897.0^a^	475.7	1596–2455
crushed tomato puree (1)	112.9^a^		
tomato jam (2)	142.1^a^	85.35	81.74–202.4
soy sauce (6)	1516^b^	446.3	285.5–3483
barbecue sauce (2)	465.2^b^	295.5	256.3–674.2
balsamic vinegar (4)	46.7^b^	39.0	22.9–104.9
pineapple juice from concentrate (4)	56.0^b^	46.3	3.0–116.0
grape juice from concentrate (3)	34.6^b^	12.6	11.3–54.4
fruit juices from concentrate (3)^d^	82.5^b^	140	0–244.2
honey (4)	64.3^a^	30.0	34.2–100.0
beer (4)	79.3^b^	49.2	36.1–141.1
sugarcane molasses (1)	308.5^a^		
dehydrated fruits (4)^e^	1.0^a^	2.0	0–4.0
raisins (3)	176.4^a^	153.6	18.4–483.5
prunes (2)	39.1^a^	29.7	18.1–60.1
dried apricot (1)	309.1^a^		
plum jam (1)	70.72^a^		
fried onion (3)	152.1^a^	163.8	30.5–338.4

a ng/g.

b ng/mL.

c No. of samples of each type.

d Multifruit, pear, and tropical fruit juices.

e Pineapple, banana, papaya, and dates.

f *X*, mean.

## 4 Discussion

The results described above have shown the isolation, chemical characterization, and mechanism of formation of perlolyrine, a βC containing a furan ring, from tryptophan and carbohydrates, and its identification and occurrence in foods. This βC has been previously isolated from a number of natural products^31,32^ and also identified as a chemopreventive agent in Maillard reactions.^35^ As described here, this βC results from the reaction of l-tryptophan with the α-dicarbonyl compound 3-DG and appears in reactions of tryptophan with glucose, fructose, and sucrose. The formation of perlolyrine occurs under acidic conditions and with the increase of the temperature. Very high temperatures (i.e., 130 °C) did not seem to enhance the formation of this compound as compared to lower temperatures (80–90 °C). Perlolyrine formation was not favored under physiological conditions (37 °C, pH 7.4). In contrast, perlolyrine was easily produced from tryptophan and carbohydrates under heating. These results suggest that the formation of this βC during food processing and cooking is remarkable.

It is known that tryptophan reacts with carbonyl compounds (e.g., formaldehyde and acetaldehyde), affording 1,2,3,4-tetrahydro-β-carboline-3-carboxylic acid (THβC-3-COOH) through the so-called Pictet–Spengler reaction.^39,40^ A subsequent oxidative decarboxylation converts these tetrahydro-β-carbolines into aromatic βCs (e.g., norharman and harman).^20−22^ In this regard, it might be assumed that tryptophan reacts with 5-HMF, a well-known degradation product of sugars, giving rise to the corresponding tetrahydro-β-carboline-3-carboxylic acids, that followed by oxidation and decarboxylation, affords the aromatic βC perlolyrine. However, as shown here, perlolyrine did not form in that way. Instead, perlolyrine resulted from 3-DG in a reaction similar to that of the α-dicarbonyl-derived βCs and carbohydrate-derived βCs.^23,28^ The proposed mechanism is described in Figure 4. The initial steps are similar to those first described for βCs derived from glyoxal and methylglyoxal and also the βCs **1ab** arising from 3-DG.^23,28^ Tryptophan reacts with 3-DG coming from carbohydrate degradation, and after a keto-enediol or imine-enamine tautomerism,^23,28^ it cyclizes to give the 3,4-dihydro-β-carboline-3-carboxylic acid intermediates bearing an OH group at the C1′ position (C_1′_-OH). This mechanism that has been first proposed for the formation of carbohydrate-derived βCs^23,28^ differs from the classical Pictet–Spengler reaction since it affords 3,4-dihydro-β-carboline-3-carboxylic acids. In this work, these intermediates were detected by HPLC-MS and, after isolation, they afforded the βC **1ab** when heated. Alternatively, the 3,4-dihydro-β-carboline-3-carboxylic acid intermediates (imines) may oxidize to the corresponding C_1′_ = O (ketoimines or α-iminoketones). Indeed, this type of oxidation of imines to ketoimines has been reported in the literature under air or oxidants such as selenium dioxide (SeO_2_).^41,42^ This oxidation could be accompanied with oxidative decarboxylation to give the aromatic βC with the C_1′_ = O moiety that could dehydrate, cyclize upon reaction with the C_4′_-OH to give the dihydrofuran ring, and dehydrate again to afford perlolyrine. The results obtained here supported this mechanism because when the HPLC fraction (4.7–6 min) corresponding to 3,4-dihydro-β-carboline-3-carboxylic acid (giving a pseudomolecular ion [M + H]^+^ at *m*/*z* 349 and with λ_max_ at 355–370 nm) was isolated and treated with SeO_2_ and heated, it afforded perlolyrine (Figure S3).

Perlolyrine occurred in the reactions of tryptophan with fructose, glucose, and sucrose under acidic conditions and heating. The formation from fructose and also from sucrose after acidic hydrolysis occurred in higher yields than from glucose. As seen above, the direct precursor of perlolyrine is 3-DG, a main α-dicarbonyl intermediate derived from the dehydration of carbohydrates, and particularly fructose.^29,43,44^ Then, 3-DG generated through the degradation of sugars will react with tryptophan, affording perlolyrine. 3-DG occurs along with other α-dicarbonyls in foods, where it is the predominant compound with concentrations up to 410 mg/L in fruit juices, 2622 mg/L in balsamic vinegars, and 385 mg/kg in cookies.^45^ 3-DG is also present in biological samples such as blood and plasma.^29,43^ It has been suggested that 3-DG and other α-dicarbonyls could be involved in cellular damage.^30,46^ They react with free amino acids and proteins, affording irreversible AGEs that might have a role in diseases such as diabetes mellitus, Alzheimer’s disease, and atherosclerosis.^43,47,48^ As shown here, 3-DG reacts with tryptophan to give perlolyrine that could be a type of AGE. Under normal physiological conditions, the formation of perlolyrine is not favored. In contrast, perlolyrine can easily form during food processing and/or cooking and, consequently, it is daily ingested during food consumption and could occur in the body similarly to other βCs.^1^

The results in this work have shown the presence of perlolyrine in many foods. It appeared in processed foods such as tomato products, including tomato juice from concentrate, fried tomato, tomato concentrate, canned crushed tomato, tomato sauces such as ketchups and barbecue sauce, and in other foods such as molasses, soy sauce, balsamic vinegar, beer, and fruit juices from concentrate juice as well as dried fruits, fried onion, or honey. The presence of this βC was confirmed by HPLC-MS, and its concentration in foods was determined by HPLC with fluorescence detection. The content of this βC varied among different foods and between different samples within the same type of food (Table 1). The highest concentrations were found in soy sauces and tomato processed products, while other samples such as beer, dried fruits, fruit juices made from concentrate juice, and honey contained moderate concentrations. The results here suggest that foods with tryptophan and carbohydrates that are processed by heating can easily form perlolyrine. Indeed, the formation of this βC occurred during food processing as proven here with fresh tomato juice and tomato puree (Figure 8). No perlolyrine was found in fresh samples in contrast with heated samples. Then, the processing conditions will determine the level of perlolyrine in foods explaining variations within samples. The presence of perlolyrine in foods like tomato products, fruit juices, or sauces that contain tryptophan and carbohydrates, and particularly fructose, supports the mechanism of formation of this compound from 3-DG generated from carbohydrates during processing and heating, which reacts with tryptophan under acidic conditions, as shown in Figure 4. The previous knowledge about perlolyrine in foods is scarce. Perlolyrine was identified in soy sauce and beer^33,34,38,49^ with concentrations up to 3.2 and 0.14 μg/mL, respectively,^38,49^ which are similar to those found here. However, the factors influencing its formation and the mechanism involved remained unknown. This work highly increases our knowledge on this βC. The levels of perlolyrine reported in Table 1 are somehow comparable to that of other βCs coming from 3-DG such as the carbohydrate-derived βCs **1–3**.^23,28^ Indeed, they arise from the same precursor and appear in the same foods (e.g., tomato products). The levels of perlolyrine are slightly lower than **1ab** in tomato products, but they are higher in soy sauce and beer.^23^ With some exceptions such as honey (particularly Manuka honey), the levels of perlolyrine in foods were generally higher than the βCs arising from the α-dicarbonyl compounds methylglyoxal and glyoxal^28^ and also generally higher than the aromatic βCs harman and norharman.^22^ Owing to its widespread presence in foods as seen in Table 1 and its formation during food processing and cooking, it can be concluded that this βC is daily ingested via foods. An estimated exposure on the basis of highly consumed foods including tomato products, sauces, juices, beer, dried fruits and vegetables, and cooked foods could account for up to several hundreds of μg of perlolyrine/person day. Ingestion of perlolyrine may also increase by consumption of natural products. Perlolyrine has been previously identified in extracts of *Codonopsis pilosula*,^32^*Tribulus terrestris* L. fruit,^31^*Lycium barbarum* L. berry,^50^ and *Nitraria tangutorum* fruit,^51^ roots of *Sophora tonkinensis*(52) and *Panax ginseng*,^53^ and extracts of *Streptomyces* sp.^54^ In this regard, perlolyrine might have occurred during processing of natural products by the reaction of carbohydrates (3-DG) with tryptophan.

The presence and formation of βC alkaloids in foods are relevant. βCs are bioactive substances that interact with CNS receptors, inhibit enzymes (MAO and kinases), and exhibit anticancer, antimicrobial, and antioxidant actions, among others.^1,2^ βCs inhibit MAO and exhibit antidepressant and neuroprotective actions.^7−12^ Some aromatic βCs such as norharman and harman are good inhibitors of MAO,^55^ whereas others like the carbohydrate-derived βCs are poor inhibitors.^23^ Some βCs have been reported as antitumor agents and DNA binders, while others have received toxicological attention as they are comutagenic in the presence of aromatic amines and can be bioactivated to the neurotoxic *N*-methyl-β-carbolinium cations.^3,21,56,57^ Regarding perlolyrine, it has been described as an antiproliferative agent against tumor cells^35^ and an inductor (chemopreventive agent) of phase II enzymes such as quinone reductase (QR)^34^ involved in chemoprotection against cancer.^58^ Exposure to perlolyrine at micromolar concentrations resulted in 1.3–4-fold increase in NQO1 protein levels.^34^ Perlolyrine activated the human vanilloid TRPV1 and ankyrin (TRPA1) receptors and had taste modification effects.^49^ Perlolyrine was identified as a major bioactive component in medicinal plants such as *C. pilosula*,^32^*Lepidium latifolium*,^37^ or *L. barbarum* L. berry extract,^50^ as an anti-inflammatory compound in *Houttuynia cordata*,^59^ and as a weak phosphodiesterase 5 (PDE5) inhibitor.^60^ The widespread presence of perlolyrine in foods indicates that it is daily uptaken in the diet, and it could potentially exert their bioactive actions in the body since this compound could be absorbed as other βCs. On the other hand, βCs derived from α-dicarbonyls like perlolyrine could be a class of AGEs.^23,28^ The formation of perlolyrine under physiological conditions seems to be limited, but its formation in foods, food processing, or cooking could serve to trap harmful α-dicarbonyl compounds, which are very reactive substances, involved in glycation.

In conclusion, 3-DG reacts with tryptophan to give perlolyrine, a bioactive βC alkaloid containing a furan ring. The mechanism of formation occurs through 3,4-dihydro-β-carboline-3-carboxylic acid intermediates that can oxidize to α-ketoimine (C_1′_ = O) and the aromatic βC ring, which after dehydration, cyclization to the dihydrofuran ring, and dehydration afford perlolyrine. The formation of perlolyrine was favored under acidic conditions and with increasing temperature. The optimal temperature was between 80 and 90 °C, whereas much higher temperatures (110 and 130 °C) decreased the formation rates. Perlolyrine was formed in the reactions of tryptophan with carbohydrates. Fructose gave higher yields than glucose, whereas sucrose afforded perlolyrine after acidic hydrolysis and heating. Perlolyrine was identified and quantified in many foods, and its formation occurred during food processing by heating. Perlolyrine is daily ingested in the diet owing to its widespread presence in highly consumed foods, and exposure to this βC is expected to increase with food cooking.

## Supporting Information Available

^1^H NMR and ^13^C NMR spectra of perlolyrine (Table S1 and Figure S1) and HPLC and HPLC-MS chromatograms (Figures S2–S5) (PDF)
