# Supplementary material for: Identification, Formation, and Occurrence of Perlolyrine: A β-Carboline Alkaloid with a Furan Moiety in Foods
Source: J Agric Food Chem. 2023 Aug 31;71(36):13451–61. doi: 10.1021/acs.jafc.3c03612 (PMC10510388; doi:10.1021/acs.jafc.3c03612)
Supplement: Supplementary file 1 — jf3c03612_si_001.pdf [file jf3c03612_si_001.pdf]

Supporting information

IDENTIFICATION, FORMATION AND OCCURRENCE OF PERLOLYRINE: A β-CARBOLINE ALKALOID WITH A FURAN MOIETY IN FOODS

Tomás Herraiz[1]*, Adriana Peña[1], Antonio Salgado[2]

[1]Instituto de Ciencia y Tecnología de Alimentos y Nutrición (ICTAN-CSIC). Spanish National Research Council (CSIC). José Antonio Novais 6, Ciudad Universitaria 28040, Madrid, Spain.

[2]Centro de Espectroscopía de RMN (CERMN), Universidad de Alcalá (UAH), Campus Universitario Ctra. Madrid-Barcelona km 33.6, 28805, Alcalá de Henares, Madrid (Spain)

Table S1. NMR data of perlolyrine isolated from tryptophan and fructose. NMR experiments were done in a Varian INNOVA NMR System (^{1}H at 499.61 and ^{13}C at 125.62 MHz). All spectra were recorded at 25ºC.

	**^{13}C δ (ppm)**	**^{1}H δ (ppm)**
C-1	133.14	-
N-2	-	-
C-3	138.18	8.37 (d, *J* = 5.1 Hz, 1H)
C-4	113.63	8.07 (d, *J* = 5.1 Hz, 1H)
C-4a	129.42	-
C-4b	120.62	-
C-5	121.63	8.26 (d, *J* = 8.2 Hz, 1H)
C-6	119.70	7.29 (ddd, *J* = 8.0, 7.1, 1.0 Hz, 1H)
C-7	128.41	7.60 (ddd, *J* = 8.4, 7.0, 1.2 Hz, 1H)
C-8	112.42	7.76 (d, *J* = 8.2 Hz, 1H)
C-8a	130.46	-
NH	-	11.19 (s, 1H)
C-9a	140.92	-
furan C-2	156.75	-
furan C-3	109.02	6.58 (d, *J* = 3.3 Hz, 1H)
furan C-4	109.62	7.21 (d, *J* = 3.3 Hz, 1H)
furan C-5	152.09	-
CH_2OH	55.94	4.67 (d, *J* = 6.2 Hz, 2H)
$CH_2O\underline{H}$	-	5.44 (t, *J* = 6.2 Hz, 1H)

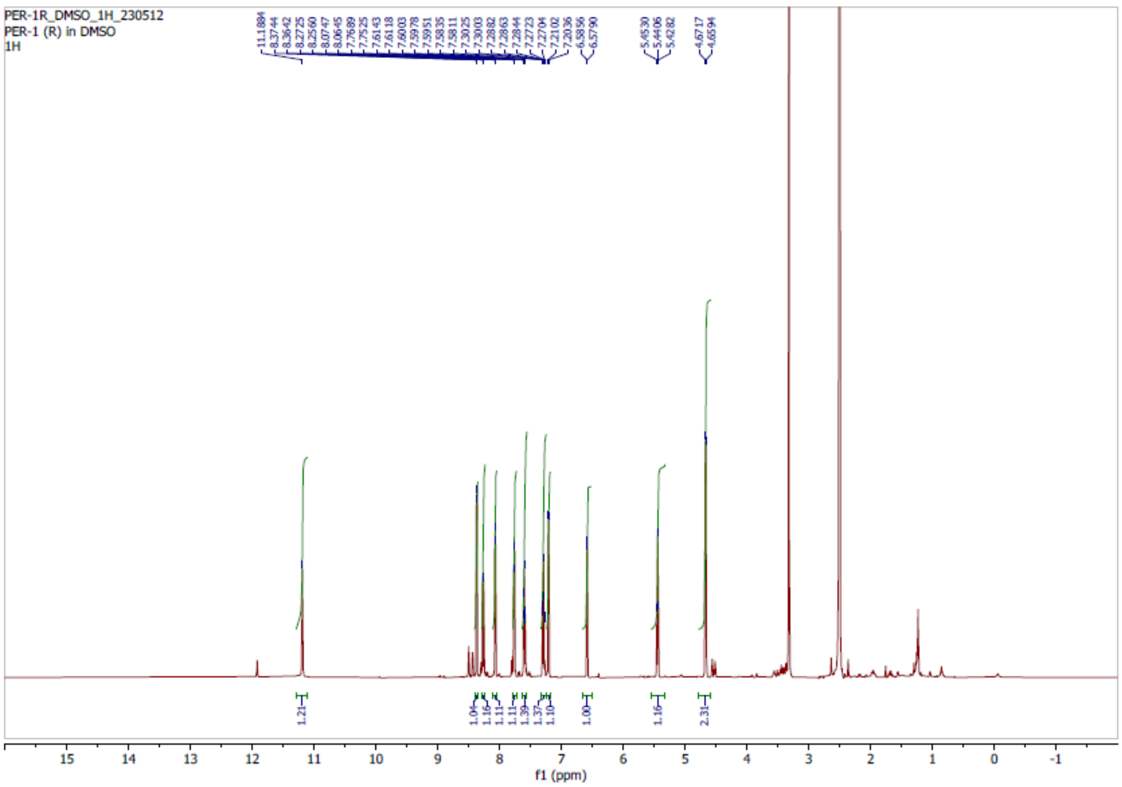

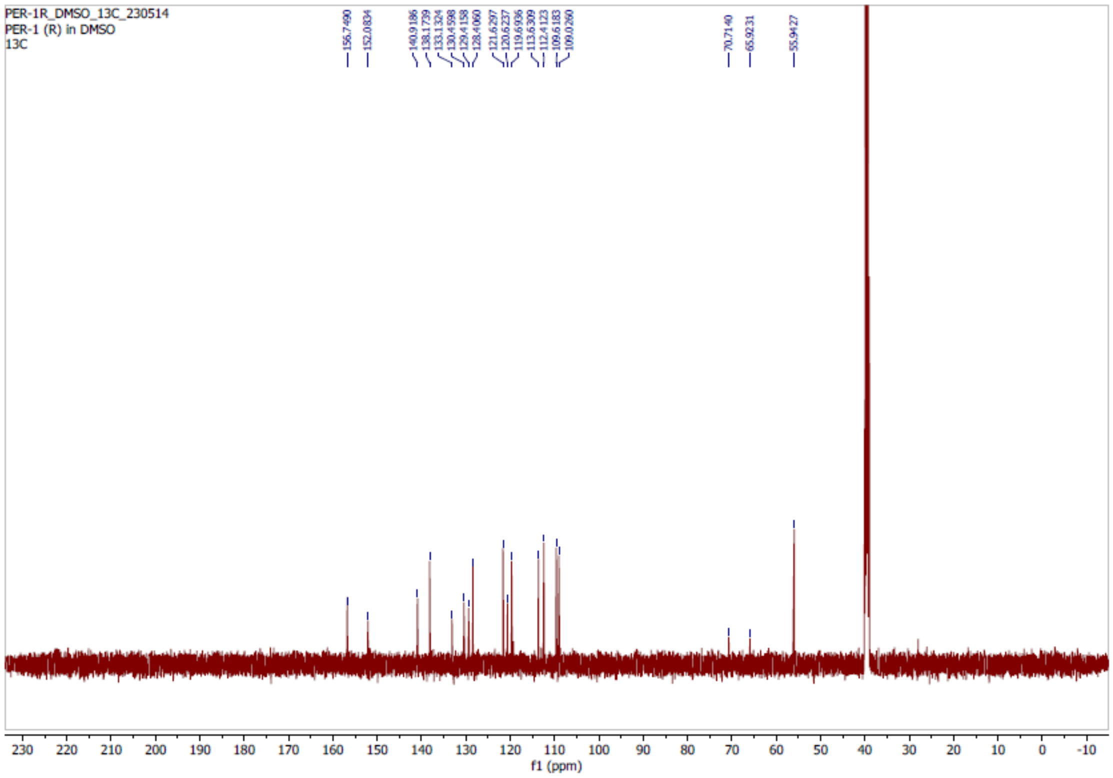

Figure S1. ^{1}H-NMR and ^{13}C-NMR spectra of perlolyrine.

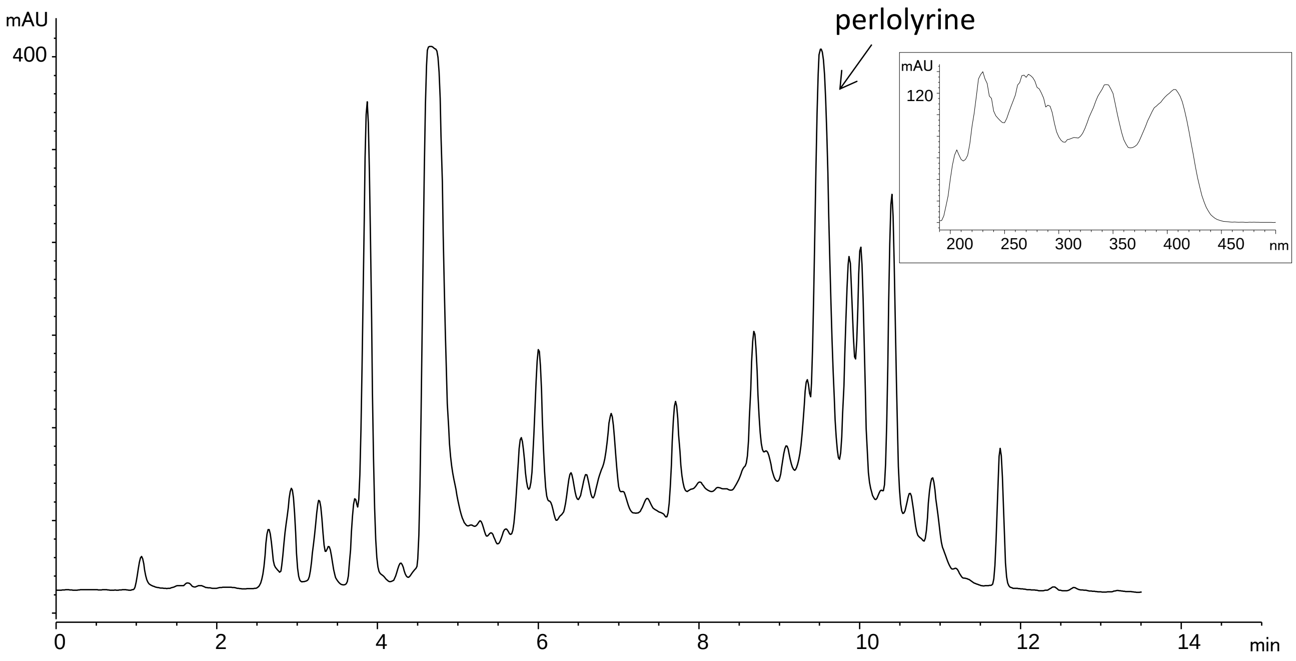

Figure S2. HPLC chromatogram (λ 280 nm) of a reaction of L-tryptophan and *D*-fructose used for the isolation of perlolyrine. DAD spectra of perlolyrine is also shown.

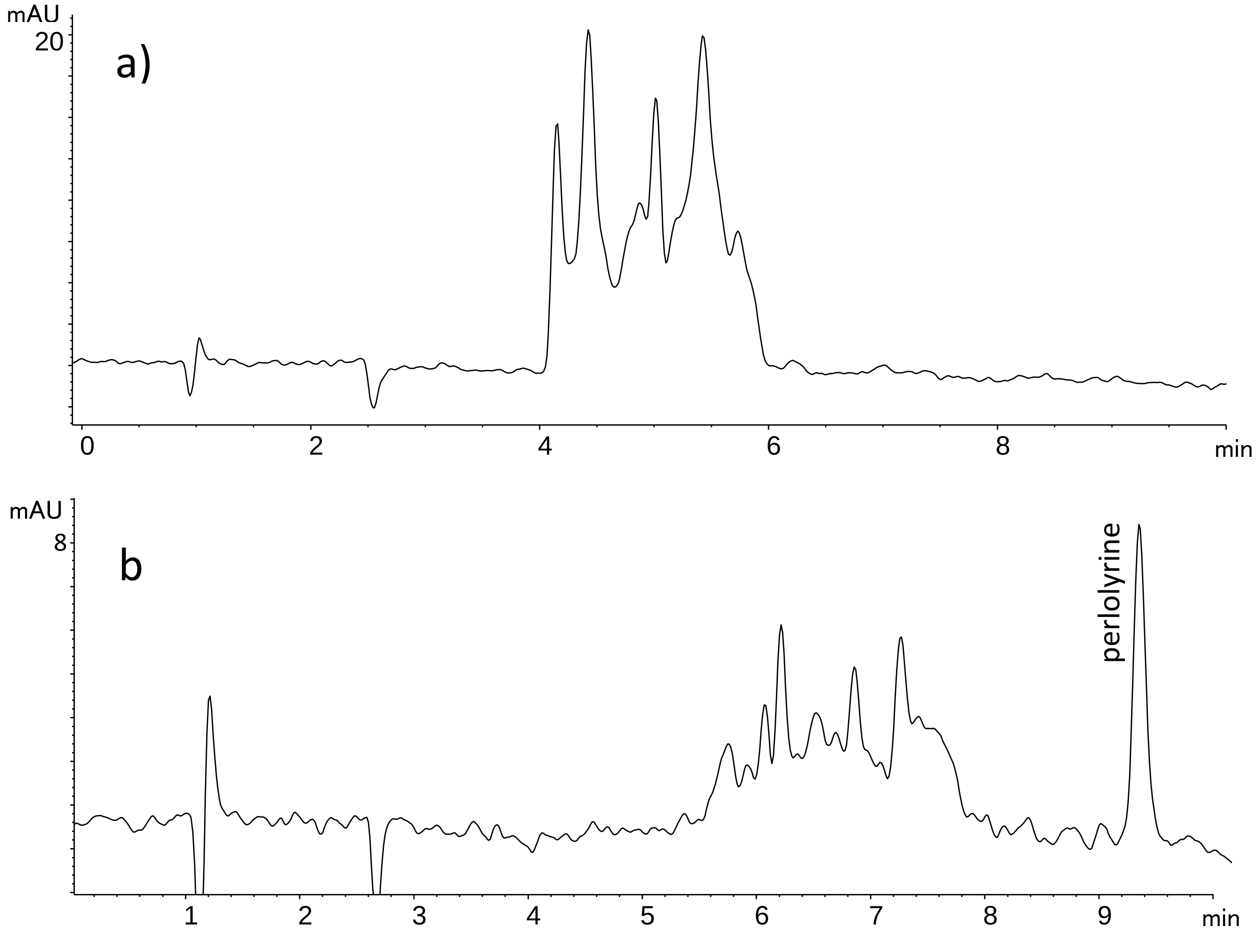

Figure S3. a) HPLC chromatogram (λ 355 nm) of the HPLC fraction containing 3,4-dihydro-β-carboline-3-carboxylic acid intermediates (absorption maxima at around 355-370 nm and $[M+H]^+$ at 349) that were isolated from successive HPLC injections of the reaction of tryptophan and preheated fructose (70ºC, 4 h). b) HPLC chromatogram (λ 355 nm) of the same fraction after treating with SeO_2 at 70ºC, 3 h.

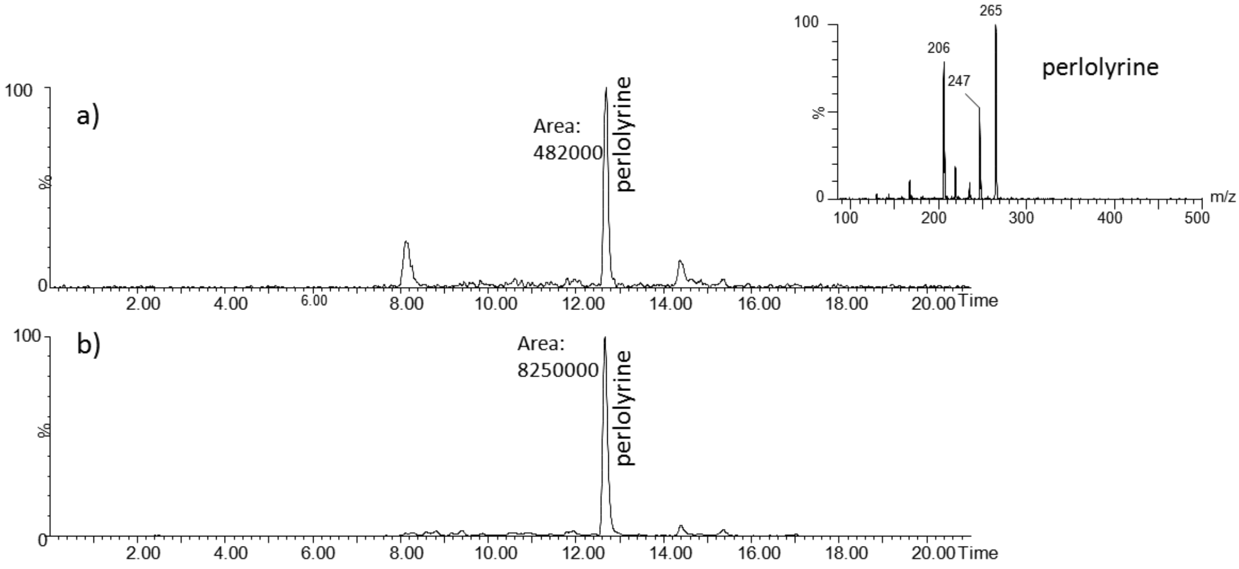

Figure S4. a) HPLC-MS analysis of tryptophan (0.5 mg/mL) reaction with glucose (5 mg/mL) (a) or fructose (4.5 mg/mL) (b) at pH 3 (80ºC, 20 h). Trace of m/z 265 $[M+H]^+$ and mass spectra of perlolyrine.

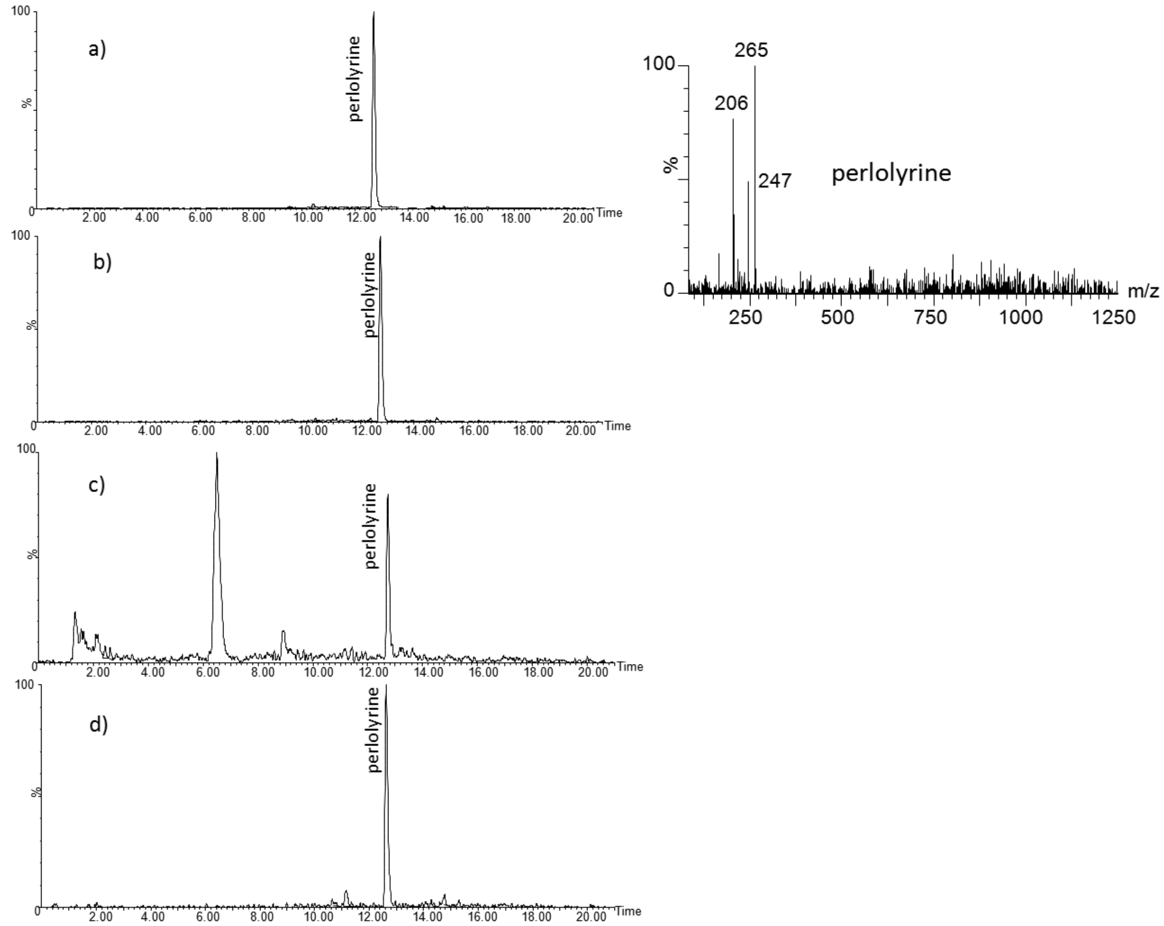

Figure S5. HPLC-MS (ESI$^+$) of foods after isolation by SPE with the *m/z* 265 $[M+H]^+$ trace and mass spectra of perlolyrine. Tomato concentrate (a), ketchup (b), beer (c) and dried apricot (d).
